# Going the Distance: Ethics of Space and Location on Accessing Reproductive Services in Australia

**DOI:** 10.1007/s11673-023-10240-z

**Published:** 2023-03-20

**Authors:** Angie Sassano, Christopher Mayes, Ian Kerridge, Wendy Lipworth

**Affiliations:** 1 Deakin University, 221 Burwood Highway, Burwood, 3125 Australia; 2 Alfred Deakin Institute of Citizenship and Globalisation, Deakin University, 75 Pigdons Rd, Waurn Ponds, 3216 Australia; 3 Bioethics and Medicine, Sydney Health Ethics, Faculty of Medicine and Health, University of Sydney, Sydney, NSW Australia; 4 Haematology Department, Royal North Shore Hospital, St Leonards, NSW Australia; 5 Department of Philosophy, Macquarie University, Sydney, NSW Australia; 6 Sydney Health Ethics, Faculty of Medicine and Health, University of Sydney, Sydney, NSW Australia

**Keywords:** Rural bioethics, Assisted reproductive technology

## Abstract

Qualitative studies on assisted reproductive technology commonly focus on the perspectives of participants living in major metropolises. In doing so, the experiences of those living outside major cities, and the unique way conditions of spatiality shape access to treatment, are elided. In this paper, we examine how location and regionality in Australia impact upon access and experience of reproductive services. We conducted twelve qualitative interviews with participants residing in regional areas across Australia. We asked participants to discuss their experience with assisted reproduction services and the impacts of location on access, service choice, and experience of care, and analysed the data using reflexive thematic analysis, as outlined by Braun and Clarke (2006, 2019). Participants in this study reported that their location impacted the services available to them, required considerable time in travel, and reduced continuity of care. We draw on these responses to examine the ethical implications of uneven distribution of reproductive services in commercial healthcare settings which rely on market-based mechanisms.

## Introduction

Rurality plays a formative role in the Australian imaginary. The valor of rural life—made up of hardworking people of steadfast character—has figured heavily in accounts of national virtue. The high standard of living experienced by those living in cities has long been characterized as dependent on the industriousness and productivity of the regions (Mayes 2019). This mythology of the bush is entangled with another prominent theme in the Australian imaginary, namely egalitarianism. This is reflected in the idea that all Australians (theoretically) enjoy universal healthcare since the introduction of Medicare in the 1980s. While these myths of rurality and egalitarianism are powerful and reinforce each other, they are not consistent with the realities of those with lived experiences of healthcare in rural areas.

Barriers and inequities in rural healthcare are well documented (Wakerman and Humphreys 2019). The Australian Medical Association’s *Rural Health Issues Survey* (AMA 2019) identified a range of issues regarding regional health, including lower access to Medicare for rural patients compared to their urban counterparts, longer wait times to see general practitioners, and poorer public facilities leading to low retention of health professionals. Similarly, the Australian Institute of Health and Welfare’s *Rural and Remote Health* (2019, ¶1) report found that those living in rural and remote areas “have shorter lives, higher levels of disease and injury and poorer access to and use of health services, compared with people living in metropolitan areas.” As others have noted, these differences between urban and regional healthcare services are exacerbated by the complexity of the healthcare system, the entanglement of public and private services, and the gradual reduction in regional public health facilities (Buykx et al. 2012). Consequently, access to health services serves as a major determinant of regional health. The issue of access is complex in health contexts (Russell et al. 2013) with inequities stemming from both *potential* access—the “opportunity to receive health care”—and *realized* access—the “utilization of health care services” (Russell et al. 2013). Both opportunity and realization of this access are differentially experienced based on geography.

Assisted reproductive technology (ART) is a case in point. Regional barriers to access to ART were recognized in the landmark Gorton review of ART in Victoria (2018a, 2018b, 2019). The review found that significant geographic barriers exist for regional people accessing ART; although providers have established clinics in major regional centres, the growing use of satellite clinics in regional towns do not always offer the comprehensive services provided in metropolitan areas (Gorton 2018a, 53). The review ultimately recommended the removal of “unnecessary . . . barriers to access,” including geographical barriers, through the provision of public facilities to improve the integrated delivery of regional reproductive services (Gorton 2019, vi). In some states, patients in rural areas are provided with travel subsidies to assist them in accessing ART services in major regional centres and metropolitan areas.

In the United States, there have been calls for interrogation of the socio-spatial components of health disparities where women must travel considerable distances or decline fertility treatment (Statz and Evers 2020). The spatial concerns regarding access to reproductive care have been further magnified in the fallout of the overturning of Roe v Wade in the United States. The resulting loss of access to abortion services in many U.S. states following the Supreme Court’s decision has as also highlighted the geographical inequities in Australia, with advocates urging for stronger actions to enable the “unfettered” access for *all* individuals, as many face a “postcode lottery” to access abortion services (Daniel 2022; Sciberras 2022). While the spatial dimensions of ART have been considered globally (Inhorn 2015), and the experiences of ART patients in metropolitan areas have received considerable attention (Waldby et al. 2013), examinations of ART access and provision have not interrogated the spatial organization of services and care, nor considered the perspectives of patients living in rural areas.

There has also been limited attention given to the effects that commercial influences have on the provision and spatial ordering of services in rural areas. This is a significant lacuna because ART is delivered almost entirely in the private sector, and commercial decisions can have significant impacts on access to care. While the for-profit model is not unique to ART, and while aspects of commercialization in ART are coming under scrutiny (Farquhar and Marjoribanks 2018; Patrizio et al. 2022), little attention has been paid to the spatial implications of the commercial organization of ART and the effects of clinics operating according to market-based logic in which the scale of patient demand determines availability.

In this paper, we attend to these significant gaps in the literature by examining how geography and regionality in Australia impact upon access to and the experience of ART services and, more specifically, how regionality and commercial interests combine to shape available reproductive services and the quality of care experienced. Our aim in doing so is to examine the ethical implications of uneven distribution of ART and provide a spatial ethical analysis of ART services in rural areas.

## Method

Twelve semi-structured interviews were conducted with individuals living in regional areas of Australia who had accessed, or were currently accessing, ART services. Five participants resided in Victoria, two in New South Wales, four in Tasmania, and one in Queensland. Recruitment was initially conducted via social media advertisements on Facebook and Twitter, with participants expressing interest by completing a short Qualtrics survey. Through this expression of interest, participants were then emailed consent information. Although this approach to recruitment garnered significant responses from individuals living in metropolitan cities, it did not attract many participants from regional locations. We therefore published a press release along with the advertisement in several Victorian regional newspapers.

We defined regionality for the inclusion criteria based on designations provided under the Australian Statistical Geography Standard, allocating regional (inner and outer), rural and remote categories to all areas outside of the major cities. Based on the demographic data, we also included participants from Hobart—the capital city of Tasmania—as part of the regional cohort due to its unique geographical conditions which make them comparative to regional centres on the mainland. An island state, Tasmania has a small population of 541,500 (Australian Bureau of Statistics 2022a); its capital city, Hobart, has a population of 238,375, which is comparative to regional locations such as Geelong with a population of 269,508 (Australian Bureau of Statistics 2022b).

Due to restrictions resulting from the COVID-19 pandemic, interviews were conducted via Zoom between April and October 2021 and lasted an average of seventy-one minutes. We asked participants to reflect on their experience of receiving ART in regional areas, specifically prompting discussion on their experience of care and service access. Interviews were designed with a standard set of questions focusing on topics such as attitudes to the organization of ART and their effects on access; ideas about improving the ART system; and issues related to innovation, evidence-based practice, advertising, and the understanding of patient as consumer. These topics were discussed in the context of participants’ personal experiences, allowing for emergence of unexpected issues and themes.

We analysed data using reflexive thematic analysis (TA) as outlined by Braun and Clarke (2019). Reflexive TA functions primarily as an inductive method whereby themes are not treated as an already existing concept within the data but rather as an “analytic output” of researcher analysis and judgement (Braun and Clarke 2006, 2019). Ongoing dialogue through a cross-analysis of the data was conducted by AS and CM to generate the core themes reported in this paper; WL and IK assisted with further interpretation. According to TA, the validity of themes and data are not reliant on “quantifiable measures” but rather with respect to their analytical value in answering research questions (Braun and Clarke 2006, 82). Therefore, reaching data saturation through an established sample size is not a requirement for meaningful reflexive TA; instead, the generation of themes accepts that “new meanings are always . . . possible” through interpretative processes which researchers choose to focus on particular patterns (Braun and Clarke 2021). Here we present the themes related to the geographical impacts of care and access to ART services.

## Themes

We developed three key themes related to the access and experience of ART services: (1) service availability and quality, (2) uneven expectations of time in travel, and (3) continuity of care.

### Theme 1: Service Availability and Quality

Service availability was a core issue reported by participants. They reflected on the impact the dispersed location of clinics, clinic closures, and the restricted service offerings had on their options and care. These concerns were often expressed in relation to the issue of choice. Veronica, for example, who lived approximately two hours from the closest major city, described how her choice in service was non-existent, as only one ART clinic was in operation in the closest regional town. While some participants, like Veronica, “chose” to use services in regional centres, others “chose” to travel to metropolitan clinics to access different services or gain second opinions. Phillipa, for example, reflected on the necessity of accessing services in the nearest metropolitan town due to the poor service availability in her region: “. . . it’s only once you’re in there you’ll learn those things, and that’s when I was—we started to question changing, going—just going to have to suck it up and go to [major city].”

Additionally, there are restricted offerings at clinics in regional areas. While Marie was located near a satellite clinic, it offered limited services, with medical imaging available only three times a week (a major issue given the time-sensitive nature of ART imaging procedures). Marie stated that:

So the . . . clinic in town is probably ten minutes from where I live . . . and you can go there for scans but they’re only open Tuesday, Thursday, Friday, I think. Which doesn’t help the scanning process. So if you need a scan on a Monday or a Wednesday you need to go to an external imaging place. Which was offered to me at one stage and I thought . . . I would rather go to [the metropolitan clinic] for a scan.

Several other participants noted restricted access to medical imaging and pathology services. Thus, in addition to there being limits to the availability of ART services, there are similarly limits to the availability of associated healthcare necessary for ART cycles.

The lack of service availability was compounded by the closure of clinics in regional towns. Several participants reported on this occurrence, perceiving the closures to be associated both with the viability of the clinic commercially and the commitment of clinicians. Sandra understood the closure of the regional satellite clinic due to the “initial [service] model [which] had it with the two local doctors and they were just struggling to maintain the service . . . it was affecting their schedule and availability . . . and didn’t suit them”; similarly, Olivia suggested that the closure of the satellite clinic in her nearby town was “because [the doctor] didn’t want to continue to travel up here.” The closure of these clinics reinforces the limits to patient choice in regional ART services, as echoed by Veronica who stated that she was left with “only one company . . . to choose.”

Interestingly, the perceived effects of service availability were not limited to the impacts on choice but also influenced the perception of quality of care by participants. Sandra compared the regional clinic with a metropolitan clinic by characterizing the former as operating in an “old Queenslander [style home]” with the laboratory “in the back”; in contrast, the metropolitan clinic was seen as “impressive . . . with all the technology in the clean clinic rooms,” which provided a different experience “particularly coming after the facility . . . [in the] Queenslander.” Veronica made similar observations by suggesting that the quality of egg retrievals was better in the metropolitan clinic as it was conducted in a hospital, compared to the regional clinic that utilized day surgery clinics. Veronica compared the clinics, stating:

. . . the egg collection and stuff, I felt was better at [metropolitan clinic company] because they did it in an hospital . . . whereas [regional clinic] . . . they do it at a day surgery place. Which—like on the other days of the week like a dentist uses day surgery. So just was a little bit sketchier than [the metropolitan hospital], it felt a bit more proper . . .

Sandra and Veronica’s comparisons demonstrate concern with both the general aesthetics of clinics and the types of facilities used, both being associated with quality.

However, there was some ambivalence around whether metropolitan service availability and quality was better than in regional setting. Sarah stated:

I guess there’s always that perception that big city, better options. You know, better specialists. Not necessarily—you know, in looking, looking statistically, I don’t think that’s necessarily the case, but you know, it’s certainly that perception that you have.

While Sarah appealed to the purported objectivity of statistics, the perception and sense that regional patients had a lower quality of care and fewer services to choose from was expressed by most of our participants. The real experience of limited service availability as well as the perception that care may be better elsewhere is ethically salient, which we discuss further below.

### Theme 2: Uneven Expectations of Time in Travel

The challenges of travelling to obtain healthcare—both ART itself and supportive pathology and medical imaging services—featured prominently in participants’ accounts. Most participants discussed the need to travel to distant metropolitan cities or regional centres to access treatments, which came with associated financial costs, including costs associated with childcare, travel, accommodation, and leave from work. Olivia, based in Tasmania, noted that, although a fifty dollar travel allowance was provided for travel to Hobart, this was manifestly inadequate as it did not fully mitigate the reality that they “still need to pay . . . the accommodation, food, time off work . . . there’s lots of expenses that add on top of it.” In addition to these expenses was the time taken to travel to metropolitan areas; Olivia mentioned that residents in the north-west of Tasmania would require a five-hour drive just to access treatment in Hobart.

While most participants were resigned to the reality of travel as something to “cop on the chin,” Sandra’s need to limit work in order to access treatment demonstrates the ethical dilemma of distance, time, and travel: “having to be able to travel and commit to IVF was a career sacrifice on my part.” Kate described how she would need to “to wipe out three quarters of the day for any . . . appointment.” Although the possibility of travel was not always perceived as negative, with some participants such as Claire seeing travel as a form of exercising patient choice and autonomy, for others, like Kate, the capacity to exercise autonomy was overridden by personal commitments.

. . . I think just realistically we just couldn’t have done Melbourne . . . it just wouldn’t have—like, we don’t have any childcare out where we are, so and you know, had a young child at home . . .

These experiences expose the complexity of normalizing travel for health and the expectation that regional patients will travel at their own costs to the metropole.

Telehealth is increasingly being adopted to minimize the burden of distance. For participants accessing treatment during COVID, the role of telehealth was seen as improving the experience of treatment by not requiring in-person consultation visits. Sarah expressed the positive benefits for the inclusion of telehealth in ART by reflecting that:

. . . initially, a couple of years ago, every appointment was in person . . . and then with COVID, now the majority of appointments are through telehealth . . . and . . . it’s almost easier

The role of telehealth is, however, complicated when treatment requires pathology, medical imaging, and surgery, as Kate observed:

I don’t know if you could do anything easier, because so—I think, do you know, the most frustrating part was all the pathology . . . you know, those days leading up to when are you going to ovulate . . . so you know, there might be, I might have done three or four days in a row travelling . . . [for] a simple . . . blood test.

Telehealth does not fully mitigate the ethical implications of time and distance. As we discuss below, it can serve to hide whose time is valuable and who is required to travel across distances. Furthermore, as Elizabeth Olson (2016, 833) argues, distance influences and “distorts” ethical assessments of who is responsible and whose interests are prioritized. Responses from participants in our study illustrate these distortions of the ethics of distance and the way patients are required to manage the distance to reproductive services or allied services such as pathology.

### Theme 3: Continuity of Care

Many participants expressed concerns about the continuity of care (the ongoing and consistent provision of care by known specialists and professionals) in the context of facility shortages and restricted service provision. The prominence of satellite ART facilities in regional locations normalized experiences of disrupted care based on the professional organization of clinics. Such satellite services often function through fly-in-fly-out (FIFO) practitioners or rotational rosters which means patients may not receive consistent treatment by one professional. This structure often leads to disrupted care, as participants would not know which clinician would be treating them. Sandra describes her experience accordingly:

. . . so there was one midwife on staff . . . and one receptionist on staff; they were local . . . she just referred me to one of the three specialists who were coming and we just went with one of them. We went to the one she—she gave it to—I’m sure she just, you know, would go down the list in rotation . . . so we went to see him, and then by the time we came to do our first pick up locally, it was a different doctor because they would come up in rotation. So I’m like, what’s the point of me going down all the way to see a particular doctor when somebody else turns out to do the treatment . . . and that was an unpleasant tone throughout the whole service.

Marie similarly shared experiences in receiving care from new consultants despite expecting chosen clinicians to be responsible for her cycle. In Marie’s experience, this led to a discomforting experience which impacted her capacity to return for some time to continue treatment due to misdiagnoses from the consultant which contradicted earlier diagnoses from the standard clinician. As she explained:

... it must have been my first—it must have been my second scan I was having, and I thought I was meeting the new consultant. I’m not a fan of ultrasounds, as many people are not, and I was quite anxious having this second one, got there and it was a different consultant and he was absolutely terrible ... he just knew nothing about me, hadn’t read anything ... and that really put me off the system because I thought I was seeing a particular doctor and then he was there

These disruptions to care were perceived to be exacerbated by resource shortages. Taylor discussed concerns with her clinic in being understaffed, leading her to need to wait significant time for results. This lack of consistent care often led some participants, such as Phillipa, to access treatments in major cities on the assumption that they would receive better resourced and more personalized care.

## Analysis

It is now broadly recognized that “place matters” in health; the dynamic social, political, historical, and environmental structures that make up place all intersect to construct particular landscapes of health (Kearns and Moon 2002). An understanding of place can assist us in critically examining the spatial organization of healthcare services and how different factors can generate inequities. In the Australian context, a critical analysis of place challenges the mythical beliefs of egalitarianism and rural exceptionalism—particularly in the context of commercialized medicine in which population concentration (i.e., market demand) determine service availability and quality, the necessity for travel, and the continuity of care. Building on the descriptive themes we have identified, we engage critically with place in the context of ART services to examine the ethical implications of distance, the spatial effects of commercialization, and the role of power in the shaping of uneven health geographies.

Distance is an accepted feature of rural life, central to the myth of rurality as existing in the “middle of nowhere” (Bourke et al. 2012; Statz and Evers 2020). Although this is normalized in regional imaginaries, and a desired feature of this lifestyle, Statz and Evers (2020) complicate this by asking “whose understanding of distance matters and when?” This question shapes the ethical dimensions of travel and service availability through geographical ideas of distance and proximity. Our analysis of the interviews suggests that it is ART companies, insurers, health bureaucracies, and specialized health professionals, rather than patients, who decide what distance does and does not matter and who dictates the spatial organization of services accordingly. Put simply, time is money, and distance is time, therefore the more time lost equates to the loss of company profit and/or clinician income. For companies it is their time and profits that matter, not the burden of distance on patients or the expenses that patients must bear to receive treatment. For participants such as Sandra who shared the need to sacrifice her career to simply have the time to travel and access treatments, it becomes clear that the burden of distance is normalized in the regional ART experience. Patients must adapt to the commercial interests which dictates where treatments are to be provided. This privileging of commercial over patient considerations is evident in the degree to which facilities are based only in locations where there is sufficient population density (i.e., market) and demographics (Danis 2008), in the “revolving door of physicians” (Cook and Hoas 2008, 55) that service rural areas and in the provision of restricted and inconsistent services by satellite clinics and via telemedicine. This highlights distance as an ethical concern that is not simply about the measurement of one point to another. Rather, distance is differently experienced and made ethically salient.

The experience of distance and its ethical salience was differentially experienced among our participants depending upon where they lived (even though all lived in regional or rural areas) and upon their access to reliable public or private transportation, care and support networks, employment flexibility, and proximity to health and other social services. For instance, Claire, who was in a position to travel for reproductive services, viewed the choice to travel as an expression of her agency. Kate, on the other hand, could not envisage travelling to Melbourne due to lack of childcare support. The ethical salience is even more pronounced when the ordering of these distances and services is largely driven by commercial interests. David Smith’s (1997, 1998a, 1998b, 1999, 2001) prolific work on moral geographies explores how ethics can reflect geographical conditions. This helps us to understand how “the where of people, how they are situated in relation to others in geographical space and place, has fundamental implications for what may be . . . right or wrong . . .” (Smith, 1998a, 9). Geo-ethical interrogations on issues such as distance and proximity thus “unbind” bioethical concepts, such as justice, from their universalizing tendencies. Resource allocation becomes a question of justice in health contexts by moving beyond the urban experience and subject as the norm (Hardwig 2006). Distance exposes the spatial expression and materialization of uneven scales of care.

The idea that ART clinics view distant environments with lower or more dispersed populations as “targets for cost-containment measures” (Hardwig 2006, 54) is evident in the spatialization of ART services across Australia, and the tendency for clinic closures to occur in regional areas. For example, in 2012, Sydney IVF closed their Northern IVF clinic in Launceston, Tasmania, due to declining patient numbers—despite a reported increase of ART treatment cycles in the state during the same period (Bryan 2011). As a result of this closure, Tasmanians were left with one ART clinic at the time, which provided comprehensive services in Hobart, and operated satellite clinics with limited services in Launceston, Burnie, and Devonport (Bryan 2011). Subsequently, the closure of their Launceston laboratory services left Hobart as the only clinic operating, “due to a lack of demand” (McLennan 2019). The implications of commercial decisions to close clinics were noted by a number of participants in the study. Olivia’s reflections on the closure of satellite services in her town due to an inability for clinicians to travel demonstrates the ways in which clinics concentrate services in metropolitan areas to minimize costs and achieve efficiencies of scale without regard to the burden placed on patients.

Commercial influence on access and experience of reproductive services is further compounded by the commercial organization of pathology services in Australia. Between 2016 and 2020, twelve pathology laboratories closed across regional Victoria (Gibson et al. 2020). By closing laboratories in smaller regional towns, companies were able to make “cost efficiencies” by consolidating services in larger regional cities or the state capital in Melbourne. If patients are unable or unwilling to travel, they may be able to have point-of-care testing with samples then being sent to laboratories in regional centres or cities. This is not an unproblematic situation, however, as noted by the Medical Scientists Association of Victoria (MSAV) which claimed that, depending on the clinical circumstances and tests performed, point-of-care testing may be “notoriously unreliable” and further complicated by “incidents of patient samples going missing or spoiling and serious delays in test results caused by transport delays” (MSAV 2018). Paul Elliot, secretary of MSAV, stated that this decline in healthcare was not acceptable purely because “private companies are making commercial decisions” (Gibson et al. 2020). In 2021, Australian Clinical Laboratories made an $88.7 million after-tax profit, it also received and kept $12 million payment from the federal government’s JobKeeper scheme (Lucas 2021). Pathology is not merely a second example of commercial forces ordering healthcare provision, but pathology is intimately connected to many ART treatments and most of our participants commented on the need to travel for pathology services. Kate, for example described the challenges of accessing pathology services as “the most frustrating part” of her ART treatment due to the need to travel for even a “simple . . . blood test.”

As with point-of-care testing, telehealth has been touted as a way to minimize the barriers to access experienced by regional patients. Some have argued that telehealth improves health outcomes for rural and regional residents through integrated and coordinated care opportunities, increased workforce retention, and the use of virtual consultations limiting the need to travel (Moffatt and Eley 2010; O’Kane 2020; Taylor et al. 2021). However, the experience of participants in this study suggests that telehealth is not a panacea for regional health. Telehealth cannot be used for physical procedures, pathology and scans, patients are still required to travel distances, and telehealth requires reliable information technology systems in order for it to function effectively. These limits were particularly salient for Veronica, who experienced a complication of ART. All consultations and appointments, with the exception of egg retrieval, pathology, and medical imaging, were done through telehealth. Following her egg retrieval procedure, Veronica experienced hyperstimulation and was unable to access the clinic for support and medical care. Ultimately, she was referred to a local emergency clinic in a nearby regional town through her obstetrician and then to a hospital not affiliated with the ART clinic.

The accounts provided by our participants reinforce the views of sociologists who have critiqued telehealth services. Using the lens of “socio-technical practice,” Warr, Luscombe, and Couch (2021) interrogate how the technological promise of telehealth is overemphasized and distorts its limited uptake due to a range of social, economic, and cultural determinants. In this context, telehealth is characterized by several “blind spots” which fail to account for different capacities of access and use (Warr et al. 2021). These blind spots often omit the effects of digital divides and exclusions, as evident in the poor network services and coverage available across regional and rural Australia, which risks exacerbating existing urban-rural health inequities. Furthermore, as noted by participants in this study, telehealth does little to address the need to access most supportive services such as pathology testing and medical imaging and can never address the need for procedures such as egg retrievals. In this way, the hype surrounding telehealth can mask and minimize the physical distances required to travel and may even be used by ART, pathology and other healthcare providers to justify closure or limitation of rural and regional services.

The accounts provided by our participants make evident that patients have limited market power in the health landscape. While all patients living in rural areas experience distance and empowerment differently depending on race, gender, class, and so on, the ART market has particular characteristics that shape the experience of patients and the commercial decisions made by clinics. Patients seeking ART services are typically healthy, young, and mobile and so are perceived as having the capacity to travel to metropolitan areas if necessary. At the same time, the extreme time-sensitivity of some ART procedures, such as egg retrieval, exacerbates the demands upon them. While, in theory, patients receiving ART may be able to exercise power and express dissatisfaction by moving between ART providers, in reality, even this power is limited in rural settings. Even where patients can choose among providers, their low numbers in rural areas means that such moves are unlikely to be of significant commercial importance. Furthermore, it is well recognized that patients will travel—even internationally—to obtain access to realize their reproductive aspirations (a phenomenon referred to by Marcia Inhorn as *reprotravel* (Inhorn 2015). This potentially provides a further rationale for clinics to centralize their services, on the assumption that patients will travel to receive them. However, as Inhorn’s work on global reprotravel shows, the financial, time, and health burdens of reprotravel is disproportionately borne by the bodies of those seeking reproductive services. Sarah, one of our participants, reflected on this burden in saying “I was wondering why I was so exhausted. And it was because I worked out that I’d spent something like thirty-eight hours in the car driving to appointments, sitting in waiting rooms, or actually having procedures done.”

In addition to creating and entrenching inequities, the concentration of ART services in cities and large regional centres and vertical integration (where clinics own support services) entrenches medical power. This power, in turn, operates spatially, which further influences patient choice and autonomy (Kenny and Duckett 2004). While the commercialization of ART is sometimes justified on the basis that a supply–demand logic enhances patient choice and patient-centricity, the experiences of patients in rural areas illustrates that it is commercial interests, rather than commitments to patient autonomy, agency, or the ideals of egalitarianism that determine the provision of services. The accounts of our participants make it clear neither respect for patients’ choices nor commitments to equity provide sufficient incentives for companies to adopt “nonfinancially lucrative” operations (Kenny and Duckett 2004, 1063). The way in which place and space operate in the commercial context therefore creates the conditions by which inequitable access becomes the norm for regional health.

Clearly, there is a recognition by government of the limitations and barriers to regional access. The concerns raised in the Gorton review and the implementation of travel subsidies are not unique to ART but demonstrate the greater strains and burdens of commercialized healthcare through a spatial lens. The lessons learnt from the ART sector, therefore, can provide valuable insights to other sections of the health system—such as medical imaging and pathology services—where similar issues of commercial operation are present in the balancing of shareholder and patient interest, access, and commercial benefit.

## Limitations

There are three key limitations to the study. First, our participant cohort was made up entirely of individuals located in regional towns across eastern Australia (Queensland, New South Wales, Victoria, and Tasmania). In contrast to central and Western Australia, which is dominated by rural and remote populations, most eastern states have a higher rate of regional ART clinics available—the Northern Territory has only one clinic in operation. Consequently, the spatial experiences of those accessing treatments in states and territories not represented might be significantly different.

Second, there is a limited engagement with intersectional barriers to access including race, class, and gender. As participation was voluntary, and advertised via social media and newspapers, there was an inevitable self-selection bias. All participants identified as cis-women, and their experiences may not reflect those of men and trans individuals in accessing ART. Unfortunately, the study also did not recruit participants from First Nations—a failure that characterizes many other empirical studies of patient experience, including in relation to ART (Boyle et al. 2020; Clarke et al. 2021; Gilbert et al. 2021).

Additionally, most participants spoke English fluently and had higher degrees and occupations in policy, science, and health-related sectors. The experiences of people from language backgrounds other than English and with lower levels of education—and presumably health literacy—might have been different. With the exception of one participant in a same-sex relationship, all other participants were solo mothers by choice or in heterosexual partnerships. Consequently, this paper is unable to engage with the intersecting and discriminatory barriers to access shared across these groups of individuals—an issue represented in the Gorton review (2019).

## Conclusion

Access is spatially experienced and materializes across geographies. Barriers to access and the normalization of distance across regional and rural locations are increasingly prominent due to a for-profit model of ART services. Understanding the geographical expressions of uneven reproductive care and broader commercial health practices helps us to see the normalization of distance from other perspectives which bring unacknowledged ethical issues into view.

Our analysis adds to the chorus of voices examining inequitable patterns of access to health services—and particularly reproductive services—in regional and rural areas. The themes of service availability and quality, time and travel, and continuity of care that emerged from our study reinforce the need for strategies to improve care for people living in rural and regional areas, including opportunities for medical training for people from regional locations and incentives for doctors to relocate to and remain in rural health centres. Our results also call for the need to problematize the market as a means for delivering equity. Rather than assuming that the market is value neutral, this study highlights the need for further exploration of geographical issues of access through the political economy of health, including greater public debate about what *truly* universal healthcare in Australia would look like and how it would be funded.

The ethical concerns raised in this paper demonstrates the potential of geography to improve and transform bioethical inquiry, and thus our theories and commitments. These commercial practices of spatializing health impact the extent of patient choice and experience of care, undermining the egalitarian ideals of health. While these issues are not unique to ART, it is a context which proves insightful in examining the inequities of access through private health settings.
